# Between forest and farmland: serological evidence of exposure to Aujeszky’s disease virus, *Coxiella burnetii* and *Chlamydia* spp. in wild boars in Poland

**DOI:** 10.2478/jvetres-2026-0045

**Published:** 2026-08-24

**Authors:** Anna Szczotka-Bochniarz, Krzysztof Niemczuk, Zbigniew Osiński, Anna Ziętek-Barszcz, Karolina Mizgier-Żuczek, Monika Szymańska-Czerwińska

**Affiliations:** 1 Department of Bacteriology and Bacterial Animal Diseases, Department of Virology and Viral Animal Diseases, Department of Research Support, National Veterinary Research Institute, 24-100 Puławy, Poland; 2 Department of Biotechnology and Nutrigenomics, Institute of Genetics and Animal Biotechnology of the Polish Academy of Sciences, 05-552 Magdalenka, Poland; 3 Department of Virology and Viral Animal Diseases, Department of Research Support, National Veterinary Research Institute, 24-100 Puławy, Poland; 4 Department of Research Support, National Veterinary Research Institute, 24-100 Puławy, Poland; 5 Office of Animal Health and Protection, General Veterinary Inspectorate, 00-002 Warsaw, Poland

**Keywords:** wild boar, Aujeszky’s disease, *Chlamydia* spp., *Coxiella burnetii*, African swine fever

## Abstract

**Introduction:**

This study assessed the seroprevalence of Aujeszky’s disease virus (ADV), *Coxiella burnetii* and *Chlamydia* spp. in wild boars in Poland.

**Material and Methods:**

A total of 1,094 serum samples were tested. Antibodies against ADV were detected by ELISA, whereas *Chlamydia* spp. and *C. burnetii* were detected using the complement fixation test (CFT). For *C. burnetii*, phase I and phase II antibodies were assessed by CFT to differentiate between long-term and recent exposure. Multiple correspondence analysis was performed to evaluate associations between seropositivity, geographical origin and wild boar population density. Serological data were integrated with environmental factors, including African swine fever (ASF) occurrence, population density and forest cover, and were visualised spatially.

**Results:**

Antibodies against ADV were detected in 455 samples (41.6%), those against *Chlamydia* spp. in 70 (6.4%) and antibodies against phase-I *C. burnetii* in 37 (3.4%) and phase-II in 30 (2.7%). Associations were observed between antibody presence, geographical location, wild boar density, pathogen co-occurrence and ASF-related environmental variables.

**Conclusion:**

Wild boars in Poland show serological evidence of exposure to ADV, *Chlamydia* spp. and *C. burnetii*, supporting their role as sentinels at the wildlife–livestock interface.

## Introduction

The wild boar (*Sus scrofa* L.) is one of the most widely distributed and rapidly expanding wildlife species in Europe (1, 14). At the interface between forested areas and agricultural landscapes, wild boars increasingly interact with domestic animals and humans. Their growing population density, ecological adaptability and movement across diverse habitats facilitate the circulation of pathogens at the wildlife–livestock–human interface (2, 11, 23).

Serological evidence of exposure to several pathogens of veterinary relevance has been reported in wild boar populations, including Aujeszky’s disease virus (ADV; suid herpesvirus 1), while serological exposure to pathogens of public health concern, such as *Coxiella burnetii* and *Chlamydia* spp., has also been reported (13, 16, 25). These pathogens were selected for this study because they represent distinct biological groups and transmission pathways and differ in their significance for animal and public health. They include a DNA virus (ADV) and intracellular or facultatively intracellular bacterial pathogens (*C. burnetii* and *Chlamydia* spp.), which rely on host cells for replication or persistence. Despite substantial differences in their biology, these pathogens share a broad host range and the ability to cross species barriers.

*Coxiella burnetii* is characterised by exceptional environmental resistance and an extremely low infectious dose, particularly in aerosol transmission, whereas chlamydiae persist primarily through intracellular infection and the ability to establish chronic or subclinical infections (10, 18, 20).

Aujeszky’s disease (AD) remains endemic in wild boar populations across Europe despite successful eradication in domestic pigs in several countries, including Poland (6). The disease’s virus exhibits strong neurotropism and can establish lifelong latency, enabling long-term persistence in wildlife reservoirs, while spillover to non-suid mammals is typically fatal (26, 29).

*Coxiella burnetii* is the aetiological agent of Q fever (17). In small ruminants, particularly goats and sheep, it is a major cause of late-term abortions and reproductive losses. In cattle, exposure to *C. burnetii* is widespread and infections are usually subclinical. In pigs, serological exposure to *C. burnetii* has been documented in both domestic and feral swine populations, although their role as a potential reservoir remains unclear (7, 8). Serological evidence of exposure to *C. burnetii* in wild boars has been reported in several European and Asian regions, suggesting that this species may act as a spillover host and contribute to environmental circulation of the pathogen (16, 17).

Chlamydial infections, including those caused by *C. suis, pecorum, C. abortus* and *C. psittaci*, have also been increasingly reported in wildlife. In wild and domestic suids, chlamydiae may be associated with respiratory, gastrointestinal or reproductive disorders, although many infections remain subclinical (10). Both *C. burnetii* and several chlamydial species have documented zoonotic potential, highlighting the importance of wildlife surveillance within a One Health framework (5, 7, 22, 25).

Although ADV has previously been monitored in Polish wild boar populations, no prior serological studies have described exposure to *C. burnetii* or *Chlamydia* spp., nor examined their combined epidemiological patterns together with ADV. This gap limits a comprehensive assessment of epidemiological risks at the wildlife–livestock interface. Therefore, this study aimed to assess serological exposure and determine the seroprevalence of antibodies against ADV, *C. burnetii* and *Chlamydia* spp. in wild boars in selected regions of Poland, and to examine associations with ecological and geographical factors, including wild boar population density, forest density and ASF occurrence.

## Material and Methods

**Wild boar samples.** The study included 1,094 wild boar serum samples collected from four provinces (voivodeships) in Poland: Małopolskie (MP, n = 50), Podkarpackie (PK, n = 38), Śląskie (SL, n = 238) and Zachodniopomorskie (ZP, n = 768). Within each province, samples originated from 1 to 17 counties (8 counties in Małopolskie, 1 in Podkarpackie, 17 in Śląskie and 7 in Zachodniopomorskie). Samples were obtained from wild boars legally hunted in 2017 by licensed hunters in accordance with national hunting regulations. As no animals were killed specifically for the purpose of this study, and all samples were collected *post mortem* during routine hunting activities, approval from an Animal Ethics Committee was not required.

The four voivodeships were selected based on results of the national AD eradication programme in domestic pigs from previous years, which had indicated difficulties in achieving disease-free status in these regions (4). These historical data (1999–2007, summarised in Table 1) were used exclusively to justify the selection of study regions and their use was not intended to reflect the epidemiological situation in domestic pigs at the time of sample collection (4).

**Table 1.** Distribution of Aujeszky’s disease in selected provinces in the years 1999–2007

Province	1999	2000	2001	2002	2003	2004	2005	2006	2007	Outbreak status
Małopolskie	+	−	+	−	+	+	+	−	−	+
Podkarpackie	−	+	+	−	−	−	+	+	−	+
Śląskie	−	−	−	−	−	−	−	+	−	+
Zachodniopomorskie	+	+	+	−	+	+	+	+	−	+

+: positive; −: negative

Molecular analyses by PCR were not performed because the study was based on archived serum samples collected within the national Aujeszky’s disease monitoring programme, which are not suitable for the direct molecular detection of the investigated pathogens.

**Serological assay for ADV.** The presence of antibodies to ADV in wild boars was determined using a commercially available ELISA for the identification of antibodies to glycoprotein E (IDEXX PRV (pseudorabies virus)/ADV gI (glycoprotein I), IDEXX Laboratories, Hoofddorp, the Netherlands), according to the manufacturer’s recommendations.

**Serological assay for *Chlamydia* spp. and *C. burnetii*.** Serological testing for antibodies against *Chlamydia* spp. and *C. burnetii* was performed using the complement fixation test (CFT) with commercially available diagnostic kits supplied by Virion/Serion (Würzburg, Germany), in accordance with the manufacturer’s instructions. Prior to testing, serum samples were diluted as required and heat-treated at 61 ± 1°C as the manufacturer’s protocol recommended. Each assay included a complete set of controls, comprising positive and negative sera, antigen controls and checks for complement activity.

For *Chlamydia* spp., serum samples were initially diluted 1 : 4, and the results were interpreted based on the degree of haemolysis inhibition observed at a serum dilution of 1 : 32; samples exhibiting ≤75% haemolysis were considered seropositive. For *C. burnetii*, antibodies against both phase-I and phase-II antigens were detected, which are interpreted as markers of long-term/past and recent exposure, respectively. A serum sample was considered seropositive when haemolysis at a dilution of 1 : 10 did not exceed 75%, whereas complete haemolysis was interpreted as the absence of detectable antibodies. No species-specific cut-off values for wild boar sera were available at the time of testing; therefore, interpretation was based on manufacturer recommendations and previous studies conducted in domestic animals.

Because the CFT primarily detects antibodies at higher titres and is less sensitive than ELISA or immunofluorescence assays, the results were interpreted as evidence of exposure rather than active infection. According to the WOAH Terrestrial Manual, the CFT is classified as a serological method that may be used for population-level surveillance and detection of immune response, despite its known limitations (27).

**Statistical analysis.** Multiple correspondence analysis (MCA) was used to identify possible associations between the presence of antibodies against ADV, *Chlamydia* spp. and *C. burnetii* in the tested serum samples and various categories of variables, including the geographical origin of the samples, wild boar density (WBD), African swine fever (ASF) occurrence (included as an ecological contextual variable rather than a directly related pathogen) and agreement patterns (both concordant and discordant) among the results for ADV, *Chlamydia* spp. and *C. burnetii* within the same serum samples. The analysis was performed using Statistica version 10.0 (StatSoft, Tulsa, OK, USA).

All variables included in the MCA were categorical. For each dimension and for each row and column point, the software calculated the standard parameters of multivariate analysis, including inertia, quality and eigenvalues. Chi-squared statistics derived from the frequency of co-occurrence of the analysed factors were used to assess the contribution of individual variables to the overall structure of the MCA model. Based on these calculations, the coordinates of the analysed categories were projected onto a two-dimensional space to generate a graphical representation of their relationships. Distances between points in the MCA plot reflect the degree of similarity or dissimilarity between categories, with closer proximity indicating stronger associations. Groups of variables forming clusters in the coordinate space were visually identified to facilitate interpretation of potential interrelationships. Samples with doubtful serological results (+/−) were excluded from the MCA analyses.

Independently of the MCA model structure, statistically significant relationships between selected pairs of categories were additionally evaluated using point G values, which were used as descriptive indicators of association strength within the MCA framework. Point G values close to zero indicated a lack of statistically significant association, whereas values greater than 0.5 were interpreted as evidence of a statistically significant relationship between the analysed categories. A probability level of ≤0.05 was considered statistically significant.

**Map visualisation.** The results of the study were compared with data on WBD and forest cover in Poland in 2017 obtained from the Forest Data Bank (3), as well as with data on ADV seroprevalence in pigs and ASF prevalence in Poland (provided by the General Veterinary Inspectorate (9) (Table 2). The geographical distribution of the results (Figs 2–5) was generated using ArcGIS 10.4.1 (Esri, Redlands, CA, USA).

**Table 2.** The results of the Aujeszky’s disease eradication programme in domestic pigs in Poland in 2017

Province	Number of tested herds	Number of infected herds	Number of tested pigs	Number of infected pigs
Dolnośląskie	2546	1	11 371	2
Kujawsko-Pomorskie	16 167	0	94 898	0
Lubelskie	18 080	0	77 825	0
Lubuskie	1440	0	11 517	0
Łódzkie	16 117	0	11 547	0
Małopolskie	7131	0	24 113	0
Mazowieckie	16 098	0	94 072	0
Opolskie	4493	0	23 416	0
Podkarpackie	7225	0	22 171	0
Podlaskie	3982	0	20 966	0
Pomorskie	6687	0	43 688	0
Śląskie	4277	0	18 003	0
Świętokrzyskie	6183	16	24 956	20
Warmińsko-Mazurskie	4245	0	40 388	0
Wielkopolskie	31 975	3	241 650	3
Zachodniopomorskie	1868	0	22 742	0
Total	148 514	20	887 223	25

## Results

The prevalence of antibodies against ADV and *Chlamydia* spp. is summarised in Table 3, and that of antibodies against *C. burnetii* is given in Table 4.

**Table 3.** Seroprevalence of ADV and *Chlamydia* spp. in wild boars

Province	Tested samples (n)	ADV +	ADV −	ADV +/−	*Chlamydia* spp.+	*Chlamydia* spp.−	*Chlamydia* spp.+/−
Małopolskie	50	15(30.0%)	34(68.0%)	1(2.0%)	1(2.0%)	46(92.0%)	3(6.0%)
Podkarpackie	38	1(2.6%)	37(97.4%)	1(2.6%)	3(7.9%)	32(84.2%)	3(7.9%)
Śląskie	238	73(30.7%)	152(63.9%)	13(5.5%)	17(7.1%)	210(88.2%)	11(4.6%)
Zachodniopomorskie	768	355(46.2%)	400(52.1%)	14(1.8%)	49(6.4%)	675(87.9%)	44(5.7%)
Total	1094 (100%)	445(40.7%)	623(57.0%)	28(2.6%)	70(6.4%)	963(88.0%)	61(5.6%)

totals may not sum to exactly 100% due to rounding; +: positive; −: negative; +/−: doubtful.

**Table 4.** Seroprevalence of *Coxiella burnetii* in wild boars

Province	Testedsamples (n)	Phase I +	Phase I −	Phase I +/−	Phase II +	Phase II −	Phase II +/−
Małopolskie	50	0(0.0%)	41(82.0%)	9(18.0%)	0(0.0%)	41(82.0%)	9(18.0%)
Podkarpackie	38	1(2.6%)	37(97.4%)	7(18.4%)	0(0.0%)	29(76.3%)	9(23.7%)
Śląskie	238	5(2.1%)	207(87.0%)	16(6.7%)	8(3.4%)	216(90.7%)	14(5.9%)
Zachodniopomorskie	768	31(4.0%)	672(87.5%)	65(8.5%)	22(2.9%)	695(90.5%)	51(6.6%)
Total	1094	37(3.4%)	950(86.8%)	107(9.8%)	30(2.7%)	981(89.7%)	83(7.6%)

totals may not sum to exactly 100% due to rounding; +: positive; −: negative; +/−: doubtful.

**ADV seroprevalence.** Aujeszky’s disease virus antibodies were detected in 455 samples (41.6%), whereas 611 samples (55.8%) tested negative and 28 samples (2.6%) yielded doubtful (inconclusive) results. The highest seroprevalence was observed in the Zachodniopomorskie province (46.2%), followed by the Podkarpackie (31.6%) and Śląskie (30.7%) provinces. The lowest seroprevalence was found in the Małopolskie Province (30.0%).

***Chlamydia* spp. seroprevalence.** Seropositivity to *Chlamydia* spp. was found in 70 serum samples (6.4%). The highest proportions were noted in the Podkarpackie (7.9%) and Śląskie (7.1%) provinces, while the lowest seroprevalence was recorded in the Małopolskie province (2.0%).

***Coxiella burnetii* seroprevalence.** Antibodies against *C. burnetii* were assessed using phase-specific serology. Antibodies against phase-I antigens were detected in 37 (3.4%) samples, and antibodies against phase-II antigens were found in 30 (2.7%) samples. Doubtful results were recorded in 107 (9.8%) and 83 (7.6%) samples for phase-I and phase-II antibodies, respectively, and were not considered seropositive. The highest proportion of samples positive for phase-I antibodies was observed in the Zachodniopomorskie province (4.0%), and the highest proportion of phase-II-positive samples was found in the Śląskie province (3.4%). No seropositive samples were detected in the Małopolskie province. Detailed results are presented in Table 4.

**Multiple correspondence analysis (MCA).** The MCA identified four distinct groups characterised by different patterns of association among the analysed variables (Fig. 1). Each group corresponded to a specific province and was defined by characteristic combinations of serological categories and epidemiological variables. Statistically significant associations observed within these groups are described below.

**Fig. 1.**
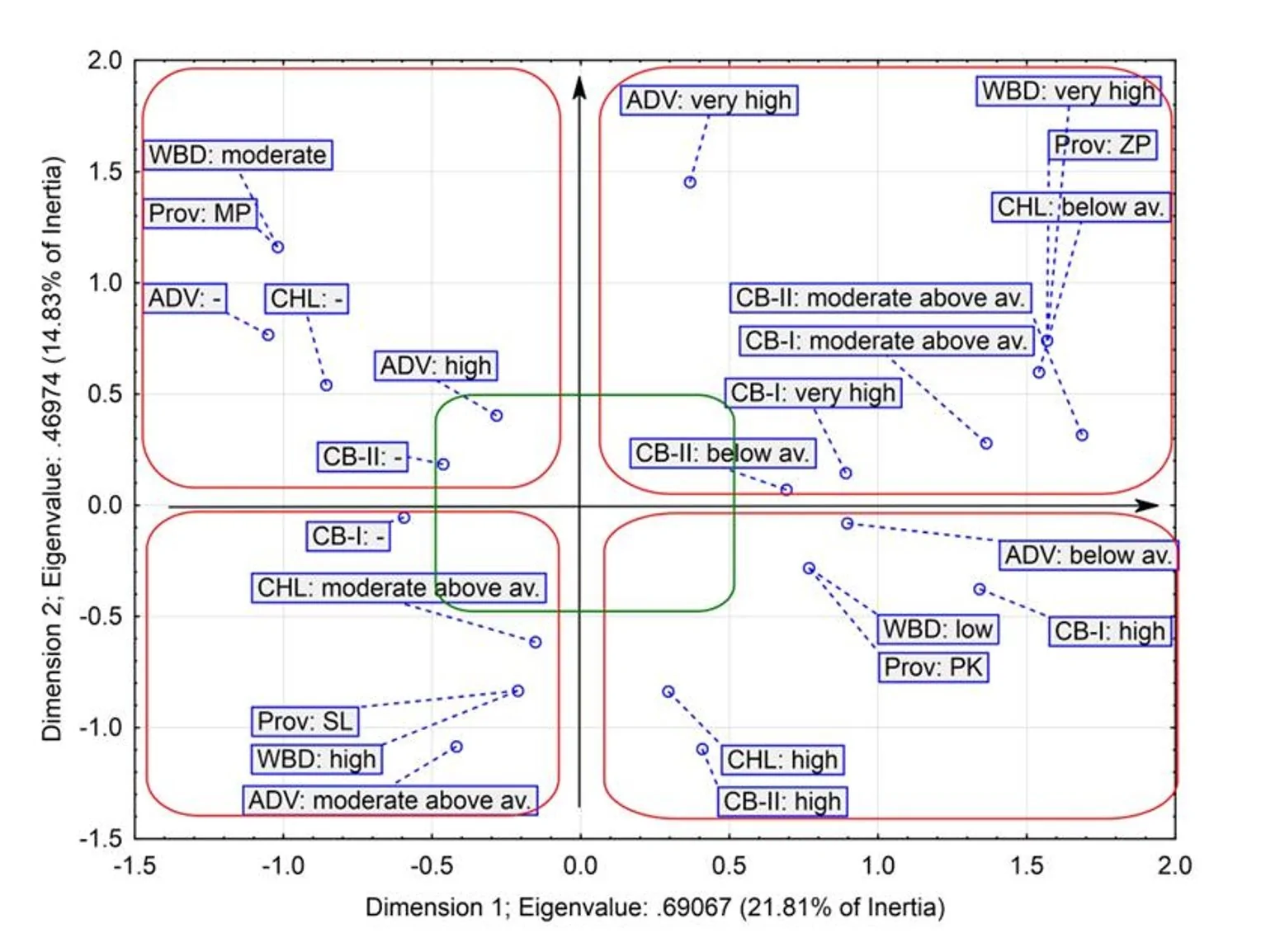
Results of multiple correspondence analysis illustrating associations between the geographical origin of wild boars, wild boar density (WBD) in individual provinces, and serological categories of antibodies against Aujeszky’s disease virus (ADV), *Chlamydia* spp. (CHL) and *C. burnetii* (CB-I for phase-I and CB-II for phase-II antigens). Locations of blue (variable-representing) points close to each other indicate stronger associations, whereas locations farther apart indicate weaker or no associations. Red (inequal quadrant) outlines – groups of categories with similar profiles; green (central) outline – variables with low discriminatory power and no clear association with other variables; av – average; Prov – province; MP – Małopolskie; PK – Podkarpackie; SL – Śląskie; ZP – Zachodniopomorskie; WBD – wild boar density

Group I (Zachodniopomorskie province), characterised by very high WBD, was associated with below-average seroprevalence of *Chlamydia* spp. and elevated levels of phase-I antibodies of *C. burnetii*, ranging from moderately above average to very high. Categories related to phase-II antibodies of *C. burnetii* showed a mixed pattern, including both below-average and moderately above-average values. Group II (Podkarpackie province), characterised by low WBD, was associated with high levels of phase-I antibodies of *C. burnetii* and below-average seroprevalence of ADV. Additionally, this group showed high levels of *Chlamydia* spp. and phase-II antibodies of *C. burnetii*, indicating a distinct serological profile despite low population density. Group III (Śląskie province), characterised by high WBD, was associated with moderately above-average seroprevalence of ADV and *Chlamydia* spp. No distinct pattern was observed for phase-specific antibodies of *C. burnetii*, which were not clearly associated with this group. Group IV (Małopolskie province), characterised by moderate WBD, was associated with low or negative serological indicators for ADV and *Chlamydia* spp. No clear pattern was observed for phase-II antibodies of *C. burnetii*, while phase-I antibodies were not strongly associated with this group.

**Map visualisation.** Based on the results of the present study and data obtained from external sources, a series of maps was prepared illustrating: (1) the spatial distribution of serological evidence of exposure to ADV, *C. burnetii* and *Chlamydia* spp. in wild boars, the occurrence of ASF and AD in domestic pigs, and WBD at the county level (Fig. 2); (2) the spatial distribution of serological markers of exposure to ADV, *C. burnetii* and *Chlamydia* spp. in wild boars in relation to forest cover (Fig. 3); (3) the spatial distribution of serological evidence of exposure to these pathogens in wild boars in relation to WBD (Fig. 4); and (4) the occurrence of AD and ASF in domestic pigs together with wild boar density (Fig. 5).

**Fig. 2.**
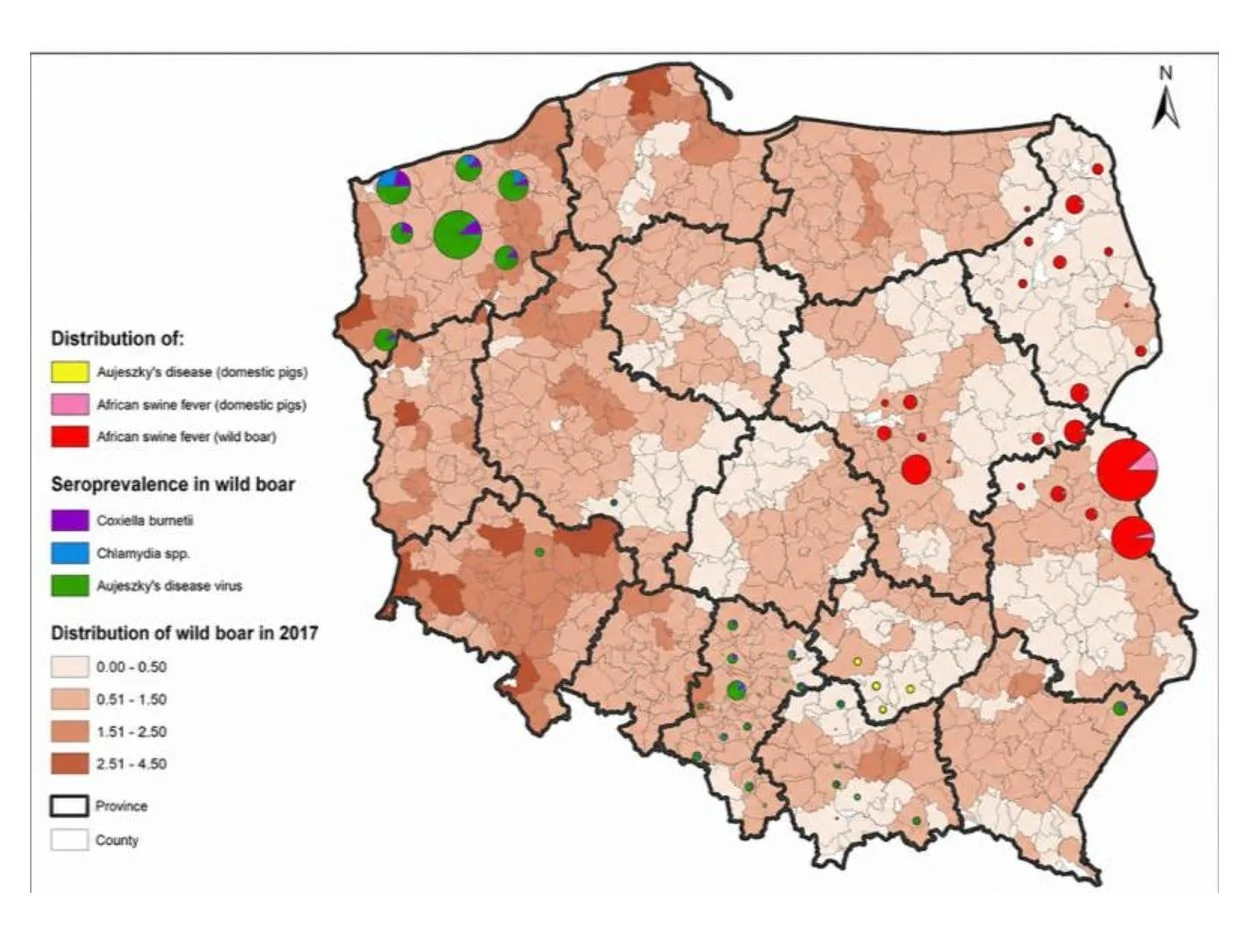
Spatial distribution of serological evidence of exposure to Aujeszky’s disease (AD) virus, *Coxiella burnetii* and *Chlamydia* spp. in wild boars, together with the occurrence of African swine fever and AD in domestic pigs and wild boar density in Poland in 2017

**Fig. 3.**
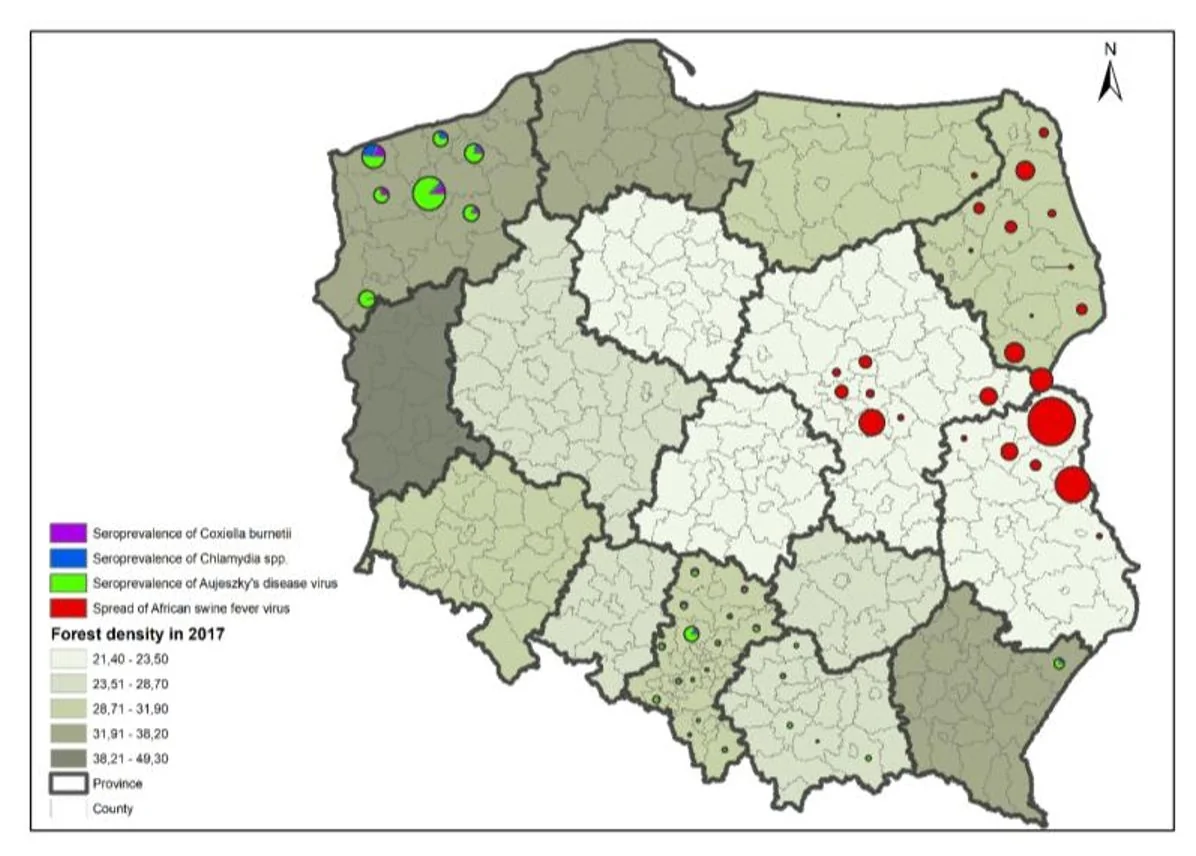
Spatial distribution of serological markers of exposure to Aujeszky’s disease virus, *Coxiella burnetii* and *Chlamydia* spp. in wild boars, shown against the background of African swine fever occurrence and forest cover in Poland in 2017

**Fig. 4.**
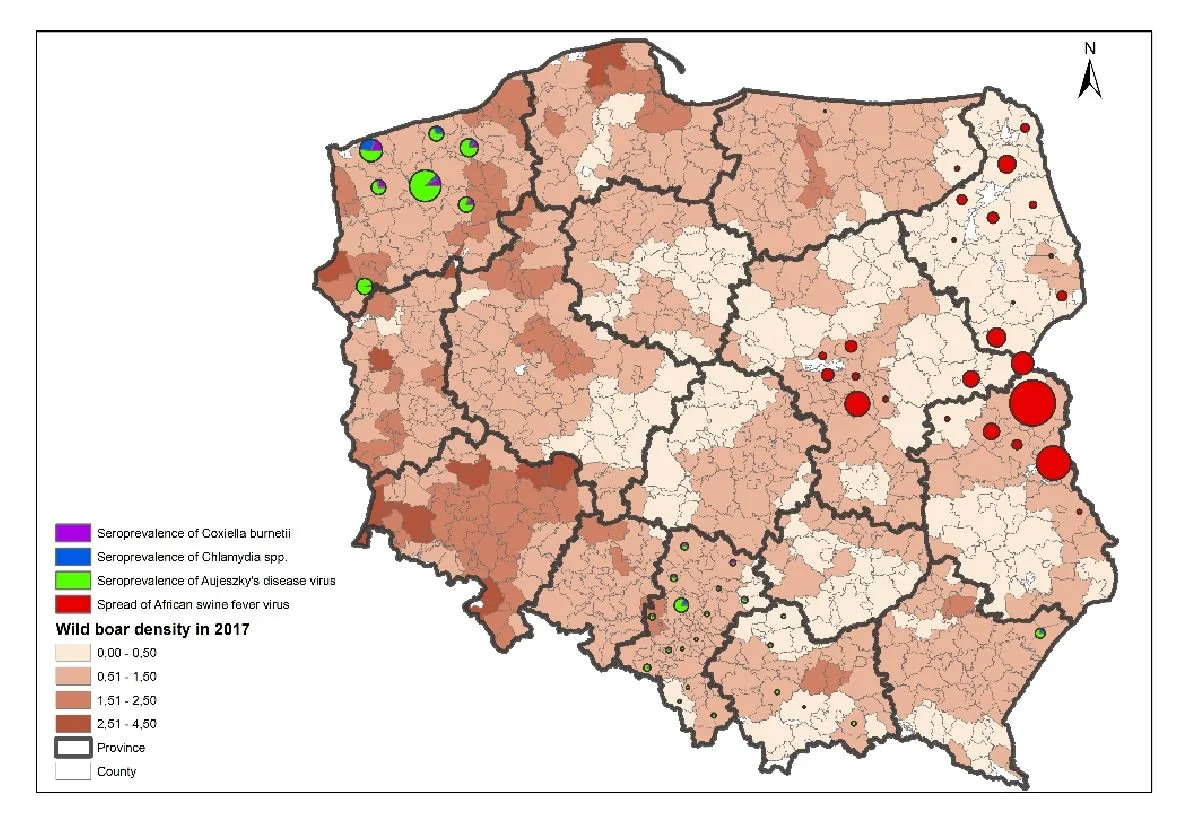
Spatial distribution of serological evidence of exposure to Aujeszky’s disease virus, *Coxiella burnetii* and *Chlamydia* spp. in wild boars in relation to wild boar density and African swine fever occurrence in Poland in 2017

**Fig. 5.**
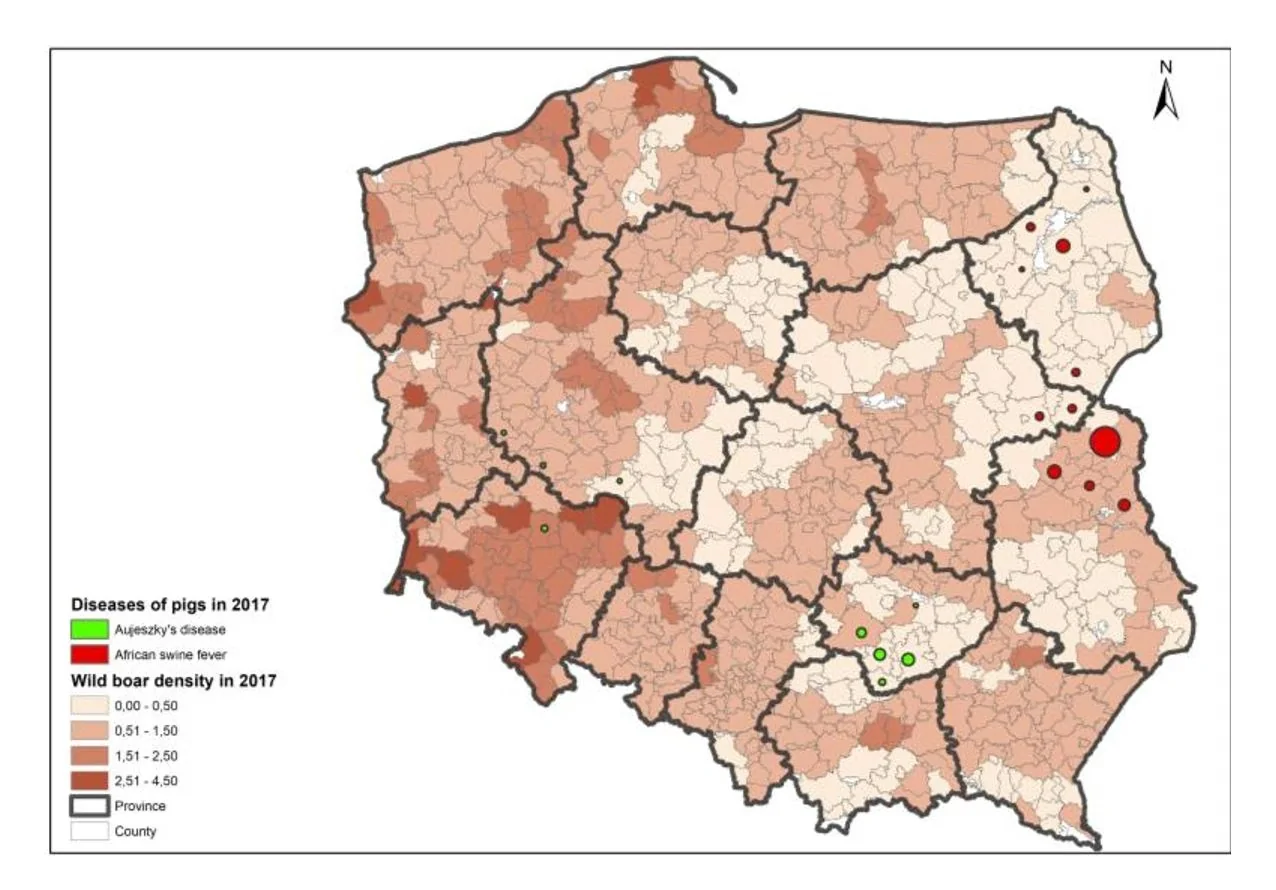
Spatial distribution of serological evidence of exposure to Aujeszky’s disease virus in wild boars, together with African swine fever occurrence in domestic pigs and wild boar density in Poland in 2017

The comparison of forest density with disease occurrence, as shown in Fig. 3, does not indicate a straightforward relationship. African swine fever occurred in wild boars irrespective of forest cover, while serological evidence of exposure to ADV, *C. burnetii* and *Chlamydia* spp. was observed in provinces differing substantially in forest density.

Figure 4 presents the spatial distribution of serological evidence of exposure to ADV, *C. burnetii* and *Chlamydia* spp. in wild boars, shown together with WBD; ASF is included as contextual information. As in Fig. 2, no direct relationship was observed between WBD and the serological indicators analysed in the present study.

The occurrence of ASF in domestic pigs was compared with WBD, and this is visualised in Fig. 5 together with the spatial distribution of serological evidence of exposure to ADV in wild boars. Higher numbers of ASF outbreaks were observed in counties with higher WBD, whereas no clear relationship was evident between WBD and serological indicators of ADV exposure.

## Discussion

The present study provides serological evidence of exposure to ADV, *C. burnetii* and *Chlamydia* spp. in wild boars from four regions of Poland. The simultaneous assessment of these pathogens offers a unique perspective on pathogen exposure at the wildlife–livestock interface, where wildlife, domestic animals and humans may interact. Although seroprevalence varied by pathogen and region, the combined findings indicate that wild boars may contribute to the maintenance of these pathogens within natural ecosystems and potentially to their transmission to domestic livestock.

Among the pathogens investigated, ADV showed the highest seroprevalence (41.6%), suggesting persistent circulation of the virus in Polish wild boar populations. These results align with earlier national monitoring, which reported an increasing trend between 2011 and 2014 (13), and are consistent with European estimates ranging from 22.2% to 76.9% (12). The highest seroprevalence observed in the Zachodniopomorskie province likely reflects its large wild boar population and extensive forest cover that enhance contact rates and viral transmission.

Importantly, none of the provinces included in this study reported ADV cases in domestic pigs at the time of sampling (6). Moreover, the spatial distribution of seropositive wild boars did not overlap with ADV-positive pig herds previously identified in neighbouring regions. This suggests that, during the study period, ADV circulation in wild boars may have occurred independently of the domestic cycle. Given the virus’s neurotropism, ability to establish lifelong latency and lethality to non-suid mammals (26, 28, 29), the continued presence of ADV in wildlife may pose a challenge to the maintenance of Poland’s ADV-free status in domestic pigs, as well as that of neighbouring countries.

Antibodies against *C. burnetii* were detected in wild boars, with phase-I antibodies found in 3.4% of samples and phase-II antibodies in 2.7%. The serological findings for *Chlamydia* spp. and *C. burnetii* should be interpreted with caution because the study design was retrospective and validated assay results for wild boars were not consultable. Moreover, as indicated in the WOAH Terrestrial Manual, the CFT mainly detects higher antibody titres and is less sensitive than ELISA or immunofluorescence assays (18, 27), potentially leading to underestimation of exposure prevalence. The relatively high proportion of doubtful results observed for *C. burnetii* may reflect limitations of the CFT assay and should be considered when interpreting prevalence estimates.

Comparable findings from Europe also show generally low seroprevalence in wild boars (16, 17), suggesting that this species may act primarily as a spillover host while contributing to the environmental circulation of the pathogen. Elevated phase-I antibody seroprevalence of *C. burnetii* in the Zachodniopomorskie province may suggest long-term circulation of the pathogen in the region, although this interpretation should be regarded as tentative in the absence of directly comparable regional data from wild boars. Conversely, the Podkarpackie province exhibited high phase-I seroprevalence despite low WBD, suggesting that factors other than wild boar abundance alone may influence exposure patterns. No serological evidence of exposure to *C. burnetii* was detected in wild boars from the Małopolskie province; however, this finding should be interpreted with caution, as it may reflect local ecological conditions, sample structure or stochastic variation rather than a true absence of exposure. These findings may also be relevant from a public health perspective, as *C. burnetii* is a zoonotic pathogen and increased seroprevalence has been reported among occupationally exposed human populations (20). The epidemiological context is further supported by studies in other wildlife species. Recent research in Poland found no evidence of exposure to *C. burnetii* in cervids (21), suggesting that wild boars, rather than large herbivores, may play a more prominent role in bridging natural and agricultural ecosystems.

The seroprevalence of *Chlamydia* spp. reached 6.4%, suggesting exposure to chlamydial agents in Polish wild boars. Comparable European studies have reported lower prevalence (25), although methodological differences between molecular and serological approaches likely account for these discrepancies.

In domestic pigs, chlamydial infections, particularly those caused by *Chlamydia suis*, are associated with conjunctivitis, enteritis, and reproductive disorders and may be associated with antimicrobial resistance determinants (10). Although infections in wild boars are often subclinical, the detection of antibodies indicates exposure and suggests that wild boars may contribute to the environmental maintenance of chlamydiae. This is particularly relevant for individuals such as hunters and butchers who handle wild boar carcasses. Chlamydial agents have been detected in wild boars (5), highlighting their ecological adaptability and broad host range. In contrast, the absence of serological evidence of chlamydial exposure in cervids in Poland (21) highlights notable interspecies differences and suggests that wild boars may be more efficient bridge hosts between forest habitats and agricultural areas.

Multiple correspondence analysis revealed distinct epidemiological profiles across the four provinces. The Zachodniopomorskie province, with high WBD, showed patterns associated primarily with elevated phase-I antibody levels of *C. burnetii.* The Podkarpackie province was characterised by high phase-I seroprevalence of *C. burnetii* despite low WBD. In contrast, the Śląskie province showed no clear pattern of association for *C. burnetii*, while moderately elevated levels of ADV and *Chlamydia* spp. were observed. The Małopolskie province, which was characterised by relatively low forest cover and included the smallest sample size, showed no serological evidence of exposure to *C. burnetii* and the lowest seroprevalence of *Chlamydia* spp.

Although ASF was not directly investigated in the present study, its inclusion on the maps was important for placing the results in the context of wild boar population dynamics (9). During the sampling period, ASF occurred primarily in north-eastern and central Poland and did not affect the regions included in this study. As ASF can substantially influence wild boar population density and movement patterns, its spatial distribution provides important ecological background for interpreting the observed patterns of serological exposure.

Collectively, these findings highlight the epidemiological role of wild boars, which may contribute to the maintenance and environmental circulation of the pathogens investigated (11). Transmission to domestic pigs may occur indirectly through contaminated straw, soil or carcasses, where *C. burnetii* can remain viable for extended periods (15, 19), facilitating indirect transmission as reported in outbreak settings (24).

The zoonotic potential of these pathogens underscores the relevance of the present results for public health, particularly for individuals handling wild boars. The study area did not encompass all regions of Poland, and serology alone cannot confirm active infection. Nevertheless, this work provides serological evidence of exposure of Polish wild boars to *C. burnetii* and chlamydial agents, offering essential baseline data and supporting the need for broader molecular surveillance.

## Conclusion

This study demonstrates exposure of wild boars in Poland to ADV, *C. burnetii* and *Chlamydia* spp., providing new epidemiological data for the two bacterial pathogens, which had not previously been documented in this species in the country. These findings support the epidemiological relevance of wild boars at the wildlife–livestock interface. Further research with broader geographical coverage and molecular diagnostic tools is needed to better characterise infection dynamics and refine risk assessments within a One Health framework.
